# Investigating Potential Associations between Cervical Procedures and HIV Acquisition

**DOI:** 10.5402/2011/789106

**Published:** 2011-11-28

**Authors:** Khady Diouf, George F. Sawaya, Stephen Shiboski, Tsitsi Magure, Rudo Makunike-Mutasa, Teresa M. Darragh, Jennifer Tuveson, Tsungai Chipato, Joel M. Palefsky, Anna-Barbara Moscicki, Michael Chirenje, Karen Smith-McCune

**Affiliations:** ^1^Department of Obstetrics, Gynecology, and Reproductive Sciences, University of California San Francisco, San Francisco, CA 94143, USA; ^2^Department of Epidemiology and Biostatistics, University of California San Francisco, San Francisco, CA 94143, USA; ^3^UCSF Helen Diller Family Comprehensive Cancer Center, University of California San Francisco, San Francisco, CA 94143, USA; ^4^Department of Obstetrics and Gynecology, University of Zimbabwe, Harare, Zimbabwe; ^5^Department of Pathology, University of California San Francisco, San Francisco, CA 94143, USA; ^6^Department of Medicine, University of California San Francisco, San Francisco, CA 94143, USA; ^7^Division of Adolescent Medicine, Department of Pediatrics, University of California San Francisco, San Francisco, CA 94143, USA

## Abstract

*Objective*. Cervical human papillomavirus (HPV) infection has been associated with human immunodeficiency virus (HIV) acquisition in populations with a high prevalence of both infections. Procedures performed in the management of cervical dysplasia may facilitate HIV entry via mechanical injury. We sought to investigate the association between cervical procedures and incident HIV. *Methods*. Data on cervical cancer screening and procedures were collected in a cohort study evaluating the diaphragm for HIV prevention in 2040 women. In this secondary analysis, we investigated the association between cervical procedures and HIV acquisition. *Results*. Out of 2027 HIV-negative women at baseline, 199 underwent cervical procedures. Cumulative risk of HIV was 4.3% over 21 months of median followup (*n* = 88). Compared with women without cervical procedures, we observed no difference in HIV incidence after a cervical biopsy (RR 0.92, 95% CI 0.39–2.16), endocervical curettage (RR 0.29, 95% CI 0.07–1.22), or loop electrosurgical excision procedure (RR 1.00, 95% CI 0.30–3.30). *Conclusions*. In this cohort, cervical procedures were not associated with HIV incidence. This lack of association could be due to the small number of events.

## 1. Introduction

Human papillomavirus (HPV) infection is one of the most common sexually transmitted infections worldwide. More than thirty subtypes of HPV can infect the anogenital tract, and specific high-risk types can lead to dysplasia and cancers of the anogenital tract in both men and women [1, 2]. It is well established that HIV-infected men and women are at increased risk of anogenital HPV infection; yet, until recently, little was known about the potential role of HPV infection in HIV acquisition. Recent studies suggest that infection with various HPV subtypes, both oncogenic and nononcogenic, is associated with increased risk of HIV acquisition in men and women [3–8].

One explanation for the association is that cervical procedures performed as part of the evaluation and treatment of HPV-induced cervical dysplasia provide a portal of HIV entry due to disruption of the normal mucosal barrier. Currently, there is little information about whether procedures performed as part of cervical cancer screening programs confer an increased risk of acquisition. In this study, we investigated whether cervical procedures performed in the evaluation of abnormal cervical cytology were associated with an increased risk of HIV acquisition in a large cohort of women in Zimbabwe.

## 2. Materials and Methods

### 2.1. Study Design and Participants

Participants were sexually active HIV-negative women enrolled in a randomized trial assessing the effect on HIV seroincidence of providing a latex diaphragm with a lubricant gel along with male condoms compared with male condom provision alone [9]. Results from the overall trial demonstrated that diaphragm/gel provision added no significant protection against acquisition of HIV [9] or sexually transmitted infections [10]; hence, data from the intervention and control arms were pooled for this study. For the parent study, women were recruited from September 2003 to September 2005; beginning in February 2004, enrollment in a study evaluating HPV outcomes [11] was offered to main trial enrollees at the Zimbabwe sites; 2040 of 2089 (97.6%) women accepted and gave written informed consent. For the present study, we performed a secondary analysis of data collected from the HPV study at the Zimbabwe sites.

### 2.2. Study Procedures

Procedures for this study are described in detail elsewhere [9]. At baseline, information on demographics and sexual behavior was collected, all participants were tested for HIV and HSV-2 using antibody testing, samples were collected for *Neisseria gonorrhea*, *Chlamydia trachomatis,* and *Trichomonas vaginalis*, and cervical cytology and an HPV swab were collected in all patients. Follow-up visits occurred every 3 months between enrollment (earliest in February 2004) and September 2006. Questions on demographics and sexual behavior were asked again, and samples were collected for HIV, HSV-2, *Neisseria gonorrhea*, *Chlamydia trachomatis*, *Trichomonas vaginalis*, and HPV. Women were treated for these coinfections if found to be positive. Methods for HPV sample collection and testing have been previously described [7]. Cervical cytology was interpreted in Zimbabwe by a single pathologist and reported using the Bethesda system [12].

### 2.3. Management of Cervical Cytology Results

**Figure 1:**
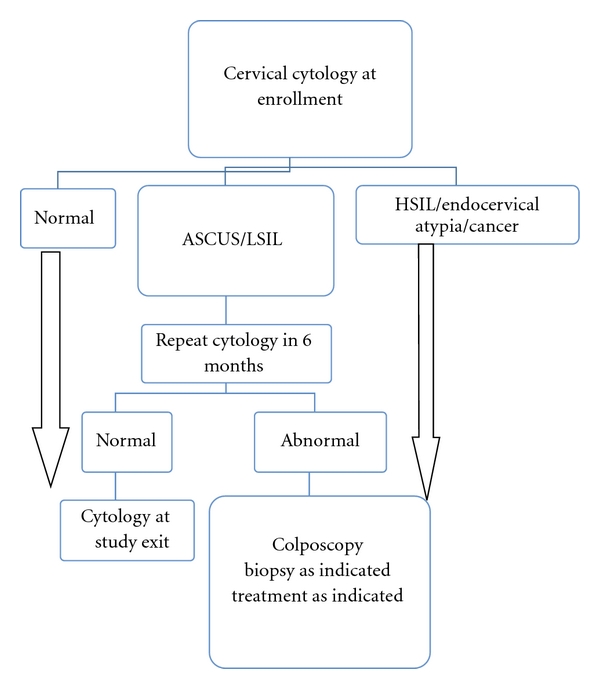
Algorithm for management of cervical cytology results.

The schema for management of abnormal cytology is shown in Figure 1. Women with a result of high-grade squamous intraepithelial lesion (HSIL), adenocarcinoma in situ (AIS), atypical glandular cells (AGC), or cancer at enrollment were referred for colposcopy. Women with low-grade squamous intraepithelial lesion (LSIL) or atypical squamous cells of undetermined significance (ASC-US) had repeated cytology in 6 months and were referred for colposcopy if that test was abnormal. All participants received a cervical cytology test at study exit and were referred for colposcopy if that test was abnormal. Loop electrosurgical excision procedure (LEEP) was performed if the colposcopically directed biopsy diagnosis was cervical intraepithelial neoplasia 2 or 3 (CIN2 or CIN3) or adenocarcinoma in situ (AIS).

### 2.4. HIV Testing

Two rapid HIV tests were performed at screening and at quarterly follow-up visits using methods described elsewhere [7]. Discordant results on rapid antibody tests were confirmed by ELISA. Once a serologic diagnosis of HIV was made, timing of HIV acquisition was assessed by RNA or DNA PCR on dried blood spots from that visit and prior visits until a negative PCR test was obtained. Date of HIV acquisition was the visit at which the first PCR test was positive.

### 2.5. Statistical Methods

We designed an *a priori *analytic plan to investigate associations between HIV incidence and cervical procedures (cervical biopsy, endocervical curettage (ECC), and LEEP). We excluded from the analysis those participants whose HIV diagnosis preceded any cervical procedure, those without an HIV test following their cervical procedures, and those with HIV tests performed less than 2 weeks after a cervical procedure (a time frame during which a test for HIV might provide a false-negative test result). In univariate analyses, we first evaluated the association between specific cervical procedures (cervical biopsy, ECC, and LEEP) and HIV acquisition. We then created a composite variable (cervical biopsy, ECC, or LEEP) and evaluated its association with HIV acquisition. In multivariate analyses, we adjusted for 3 baseline variables that had previously been shown to be significant predictors of HIV acquisition: high-risk partner, positive HSV serology, and condom use (sometimes/always versus never) [7]. In a separate exploratory analysis, we evaluated the association between timing of cervical procedures and HIV acquisition. We postulated that within a 3-month period following any cervical procedure, women were at increased risk of HIV due to mechanical damage to the cervix compared to women who had had cervical procedures >3 months ago and those who had never had a cervical procedure (reference group). The model was controlled for baseline variables defined a priori as above, specifically partner risk, HSV status, and condom use. We used the Cox proportional hazards model to analyze the association between timing of cervical procedures and HIV acquisition (STATA 10 for Windows).

## 3. Results

### 3.1. Characteristics of the Study Population

**Figure 2:**
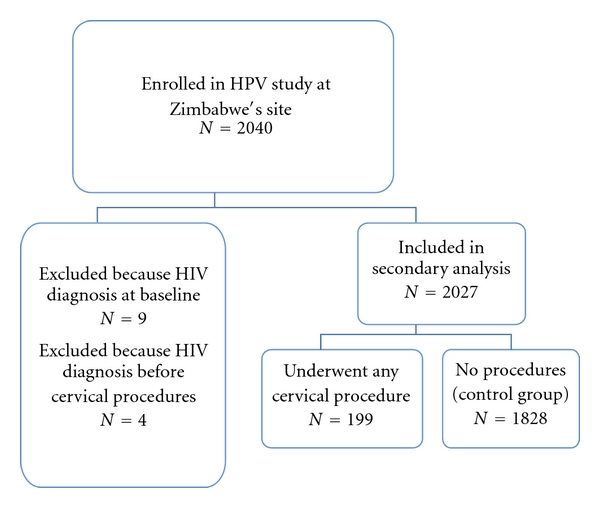
Trial profile: HPV study.

Of the 2089 women approached, 2040 women (97.6%) consented to participate in the study. We excluded 13 women from this analysis: 9 women who were HIV positive at baseline and 4 with an HIV diagnosis preceding cervical procedures (Figure 2). Therefore, this analysis was based on a total of 2027 participants, of whom 1932 (94.7%) had complete follow-up data. The median follow-up time was 21 months (range 12–24 months), and the cumulative HIV incidence was 4.3% (*n* = 88).

A detailed summary of the participants' baseline demographic and biological characteristics has been previously reported [7–10].  Table 1 presents a brief summary. The mean age of study participants at enrollment was 27 years (range 18–49 years). Most of the women reported living with a regular partner. The mean number of lifetime sexual partners was low at 1.3 (range 1–20). Approximately 7% of participants had *gonorrhea*, *Chlamydia*, *Trichomonas vaginalis*, or syphilis at baseline. All identified coinfections were treated, and screening was repeated at 3-month intervals. Approximately 50% of women had serologic evidence of prior exposure to HSV-2. Prevalence of HPV infection at baseline was 24.5%.

A total of 1674 women had normal cervical cytology at baseline (82.2%). The remainder had ASC-US (*n* = 72, 3.5%), LSIL (*n* = 102, 5%), HSIL (*n* = 12, 0.6%), AGC (*n* = 13, 0.6%), or other cytologic abnormalities (*n* = 75, 3.7%). There were 89 unsatisfactory cytologic tests (4.4%), and 3 missing results (0.15%).

There were 199 women who underwent one or more cervical procedures (Figure 2), of whom 138 had at least one cervical biopsy, 141 had an ECC, and 64 underwent LEEP. Of the participants included in our analysis who acquired HIV, 7 had undergone one or more cervical procedures.

### 3.2. Association of Cervical Procedures with Risk of HIV Acquisition

Our *priori *logistic regression model examined the association between cervical biopsy, ECC, and/or LEEP on risk of HIV acquisition; the results are summarized in Table 2. We found no statistically significant association between cervical procedures and HIV acquisition. The results were similar when adjusted for baseline variables associated with HIV acquisition (positive HSV serology, having a high-risk partner, and always or sometimes using condoms).

In a separate analysis, we examined whether cervical procedures performed within 3 months increased the risk of HIV acquisition compared to no procedures or procedures >3 months ago. This analysis used a Cox proportional hazards model to investigate the association between procedure within the last 3 months and HIV acquisition. Only 2 participants acquired HIV within 3 months of a cervical procedure. We found that women with recent cervical procedures did not have a statistically significant increased likelihood of HIV acquisition compared with women without cervical procedures in this interval (Table 3).

## 4. Discussion

This pilot study provides a new insight into the risk of HIV acquisition associated with mechanical injury to the cervix due to procedures related to cervical cancer screening. Our results show no relationship between cervical procedures and risk of HIV acquisition; however, they need to be interpreted cautiously for several reasons.

Of the 199 women who underwent cervical procedures, only 7 women acquired HIV, and, among women who had a recent cervical procedure (within the last 3 months), only 2 acquired HIV. This low number of outcomes can certainly explain our negative findings.

Our study was hypothetically subject to confounding given that all the women who underwent cervical procedures were counseled to abstain from sex for 4 weeks following cervical treatment; therefore, behavior modification could have resulted in decreased exposures to HIV during a potential period of susceptibility. However, any difference in sexual behavior between the two groups would have been limited to the first month following the cervical procedures.

There are limited data in the literature reporting the effect of cervical procedures on the risk of HIV acquisition. The effect of cervical procedures (cryosurgery, LEEP, and cone) on genital tract shedding and HIV transmission was investigated in a study by Wright et al. in 2001 [13]. This study showed greater than 10,000-fold increased local viral replication and genital shedding which persists for up to 4 weeks following treatment. This increased genital shedding, thought to be due to cervical inflammation leading to local activation of CD4 lymphocytes, could translate into a significantly increased risk of HIV transmission for partners of HIV-infected women. In HIV-negative women, cervical inflammation due to cervical procedures could theoretically increase the number of HIV target cells and lead to increased susceptibility to HIV acquisition.

Table 1 shows a protective, though not statistically significant, effect of endocervical curettage on HIV acquisition. It was our hypothesis that any cervical procedure leading to cervical inflammation may lead to increased risk of HIV acquisition. Therefore, it is unclear how to explain the protective trend shown between endocervical curettage and HIV acquisition, although the small numbers of outcomes of HIV acquisition must be considered.

There are several strengths to our analysis, including a large sample size, a low rate of loss to followup, and frequent followup. Limitations include a small number of HIV acquisition events, which affected the power and statistical precision of our findings. There were seven outcomes (HIV acquisition) in total among the 199 women who underwent procedures in this exploratory analysis. Larger studies need to be performed to confirm our findings such that we can be assured that procedures associated with cervical cancer screening and management are safe for women in areas with high endemic HIV rates.

## 5. Conclusions

This pilot study suggests that cervical procedures are not associated with an increased risk of HIV acquisition: results are not meant to be definitive given the low number of outcomes. We hope this investigation prompts larger studies on the association between cervical procedures and HIV acquisition in women, especially in areas of high HIV prevalence.

## Figures and Tables

**Table 1:** Baseline sociodemographic characteristics, reproductive history, sexual behaviors, and clinical characteristics (*N* = 2040).

Characteristic		Number	Percentage
Age	24 years or younger	758	37.2%
	25 to 34 years	937	45.9%
	35 years or older	345	16.9%

Tested positive for *herpes simplex virus*-2 serology		1034	50.8%

High-risk partner: at least one indicator^1^		1343	65.9%

Frequency of condom use in the past 3 months	Never	615	30.1%
	Sometimes	876	43%
	Always	548	26.9%

Normal cervical cytology at baseline		1674	82.3%

^1^Indicators include having any sexual partners test positive for HIV, suspect or knowing that regular partner had other sex partners in the last 3 months, ever had vaginal sex when partner was under influence of drugs/alcohol in the last 3 months, or regular partner was away from home for 1 or more months.

**Table 2:** Effect of cervical procedures at any time on risk of HIV acquisition.

Variable	HIV acquisition *n*/*N* (%)	Unadjusted relative risk (95% CI)	*P* value	Adjusted* relative risk (95% CI)	*P* value
Cervical biopsy No biopsy	6/142 (4.2) 78/1885 (4.1)	1.02 (0.43–2.38)	0.96	0.92 (0.39–2.16)	0.85

ECC No ECC	2/137 (1.4) 82/1890 (4.3)	0.32 (0.07–1.34)	0.12	0.29 (0.07–1.22)	0.09

LEEP No LEEP	3/64 (4.7) 81/1963 (4.1)	1.14 (0.35–3.71)	0.83	1.00 (0.30–3.30)	0.99

Any procedure No procedure	7/199 (3.5) 77/1828 (4.2)	0.82 (0.37–1.82)	0.64	0.74 (0.33–1.65)	0.47

CI indicates confidence interval, ECC: endocervical curettage, LEEP: loop electrosurgical excision procedure.

*Adjusted for the following baseline variables: positive serology for HSV-2, high-risk partner as defined in Table 1's footnote, sometimes or always using condoms (compared to no condom use) within the last 3 months.

**Table 3:** Effect of any cervical procedure within the last 3 months on risk of HIV acquisition^§^.

Variable	Unadjusted hazard ratio (95% CI)	*P* value	Adjusted* hazard ratio (95% CI)	*P* value
Procedure within 3 months	2.64 (0.64–10.8)	0.177	2.27 (0.55–9.36)	0.253

CI indicates confidence interval.

^§^Comparison group: no procedure ever and procedure >3 months ago.

*Adjusted for the following baseline variables: positive serology for HSV-2, high-risk partner as defined in Table 1's footnote, sometimes or always using condoms (compared to no condom use) within the last 3 month.

## Abbreviations

ASC-US: Atypical squamous cells of undetermined significance
LSIL: Low-grade squamous intraepithelial neoplasia
HSIL: High-grade squamous intraepithelial neoplasia.
